# Mavacamten optimizes myocardial work in patients with obstructive hypertrophic cardiomyopathy: a non-invasive pressure–strain analysis

**DOI:** 10.1007/s00392-026-02855-0

**Published:** 2026-02-19

**Authors:** S. Scholtz, C. Coppée, K. Mohemed, M. Potratz, F. Langkamp, V. Rudolph, C. Maack, W. Scholtz, V. Sequeira, J.-C. Reil

**Affiliations:** 1 Ruhr University Bochum, Heart- and Diabetescenter NRW, Department of General and Interventional Cardiology/Angiology, Bad Oeynhausen, Germany; 2 Comprehensive Heart Failure Center (CHFC), University Clinic Würzburg, Würzburg, Germany

**Keywords:** Hypertrophic cardiomyopathy, Mavacamten, Strain analysis, Myocardial work, Energetics

## Abstract

**Background:**

Mavacamten is the first approved myosin inhibitor for symptomatic obstructive hypertrophic cardiomyopathy (oHCM), addressing hypercontractility and left ventricular outflow tract (LVOT) obstruction.

**Objectives:**

This study evaluates left ventricular performance by non-invasive measurements of pressure–strain loops in patients treated with Mavacamten.

**Methods:**

In 36 symptomatic oHCM patients, pressure–strain analysis was performed prior to 3 and 12 months after Mavacamten therapy. Echocardiographic measurements included LVOT gradient, left ventricular ejection fraction (LVEF), global longitudinal strain (GLS), left atrial strain (LAS), peak strain time dispersion (PSD), and myocardial work parameters (global work index (GWI), global constructive work (GCW), global wasted work (GWW), and global work efficiency (GWE)). Clinical status was evaluated using the New York Heart Association (NYHA) class and stress biomarkers (NTproBNP and high-sensitivity troponin I).

**Results:**

Mavacamten therapy significantly reduced LVOT gradients at rest and under provocation. Gradients decreased from 69 ± 36 to 24 ± 27 mmHg (*p* < 0.001) at 3 months and further to 11 ± 6 mmHg (*p* = 0.003) at 12 months. Provoked gradients decreased from 113 ± 33 to 50 ± 31 mmHg (*p* < 0.001) at 3 months and to 31 ± 19 mmHg (*p* = 0.01) at 12 months. Clinical symptoms also improved. LVEF was 68 ± 6% at baseline and decreased mildly to 62 ± 5% (*p* = 0.003), while GLS and LAS remained unchanged. PSD decreased mildly from 116 ± 56 to 97 ± 36 ms and further to 93 ± 38 ms, but this was not statistically significant (*p* = 0.07). Under Mavacamten, GWE remained stable. In contrast, GWI, GCW, and GWW decreased significantly from baseline to 3 months (GWI, 2098 ± 700 to 1610 ± 440 mmHg%, *p* < 0.001; GCW, 2514 ± 776 to 1951 ± 466 mmHg%, *p* < 0.001; GWW, 312 ± 163 to 249 ± 177 mmHg%, *p* = 0.003), with only mild, non-significant further reductions at 12 months (1538 ± 402, 1901 ± 380, and 207 ± 124 mmHg%, respectively; *p* = 0.67, *p* = 0.74, *p* = 0.30).

**Conclusion:**

Myocardial work indices derived from non-invasive pressure–strain analysis were feasible to obtain in patients with oHCM in this study. Mavacamten therapy decreases workload index, constructive and wasted work, and synchronizes myocardial contractility, reflecting normalization of myocardial energetics. These findings reinforce the role of Mavacamten as a targeted therapy in oHCM.

**Graphical Abstract:**

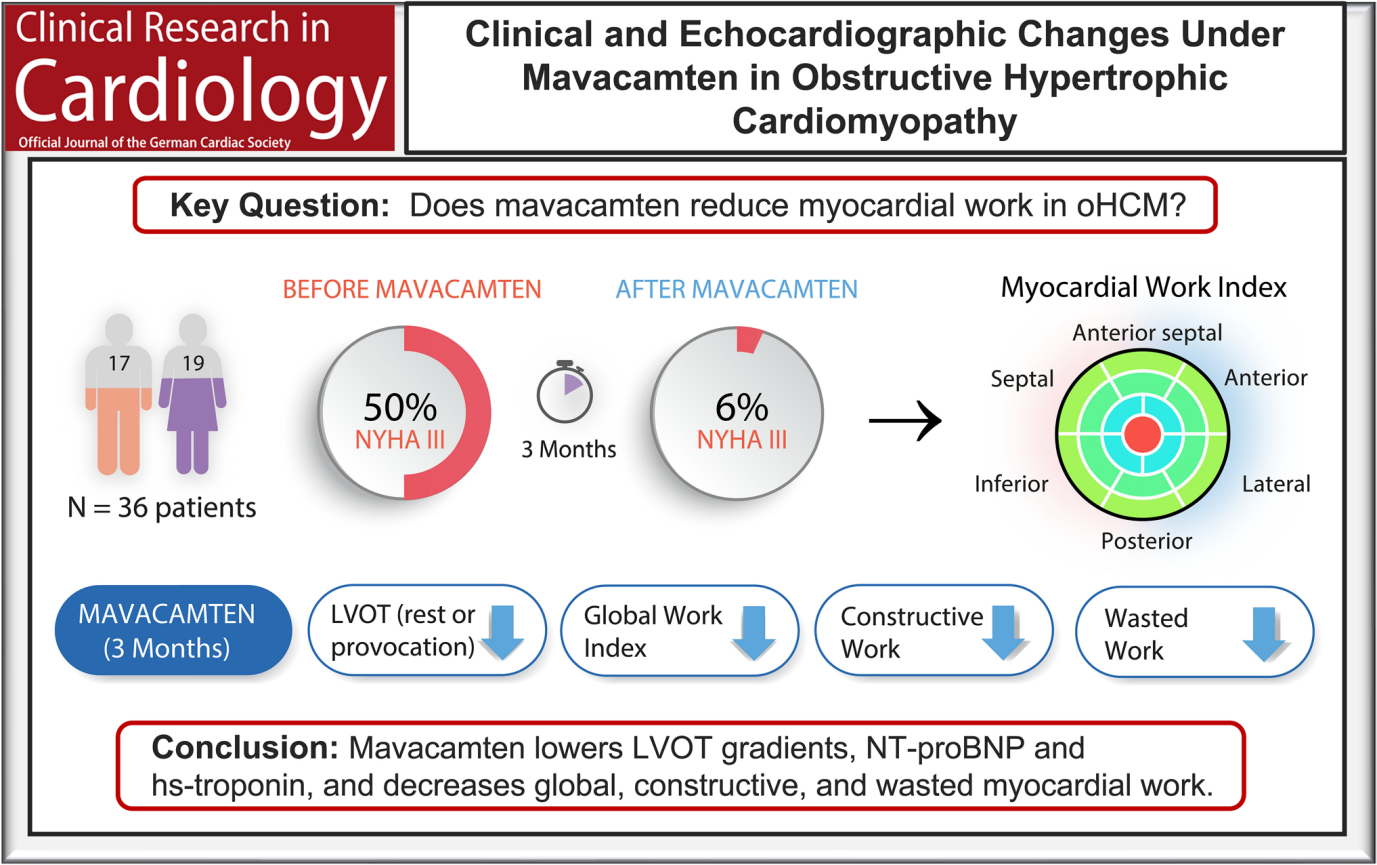

## Introduction

Hypertrophic cardiomyopathy (HCM) is characterized by left ventricular (LV) hypertrophy, hyperdynamic systolic function, and LV outflow tract (LVOT) obstruction in 60–70% of adult HCM patients, either at rest or when provoked [1, 2]. Patients with HCM and LVOT obstruction are referred to as obstructive HCM (oHCM) [3]. Dynamic LVOT obstruction imposes a significant pressure load on the LV, which activates the Anrep effect, a compensatory increase in contractility in response to raised afterload [4, 5]. While this mechanism helps maintain stroke volume and cardiac output in the face of obstruction, it comes at a significant energetic cost and mechanical inefficiency [4]. In oHCM patients, the chronic activation of this afterload-induced hypercontractility leads to excessive myocardial work for a given cardiac output, contributing to symptoms (angina, dyspnea) and disease progression through energetic inefficiency, elevated oxygen consumption, worsening diastolic dysfunction, secondary mitral regurgitation, and increased risk of heart failure and sudden cardiac death [6–8].

Current guideline-directed therapy for symptomatic oHCM focuses on reducing hypercontractility and LVOT gradients. First-line treatment includes β-blockers or non-dihydropyridine calcium channel blockers (e.g., verapamil) to blunt contractility; if symptoms persist, disopyramide or Mavacamten is recommended, with septal reduction therapies (surgical myectomy or alcohol ablation) reserved as third-line options [2].

Mavacamten is a selective inhibitor of cardiac myosin that directly targets the underlying pathology of HCM (i.e., sarcomeric hypercontractility) [9]. By shifting myosin heads into a super-relaxed (non-actin-binding) state, Mavacamten reduces excessive actin-myosin cross-bridge formation and lowers energy consumption, thereby dampening hyperdynamic contraction [10]. This mechanism-based therapy addresses the root cause of oHCM’s outflow obstruction and high energy demand, rather than only alleviating symptoms. Evidence from phase 2 and phase 3 clinical trials [11, 12] (EXPLORER-HCM and VALOR-HCM) has confirmed that Mavacamten improves symptoms, exercise capacity, reduces LVOT obstruction in symptomatic oHCM, and biomarkers of cardiac wall stress and injury, leading to its regulatory approval in the USA (2022) and Europe (2023) for this indication. However, whether it reverses the energetic inefficiency characteristic of oHCM by modulating myocardial work has not been established.

Despite preserved or supranormal LV ejection fraction (LVEF) in HCM, systolic function is not truly “augmented” in a beneficial sense—much of the contractile effort is expended as work to overcome outflow tract obstruction and increased wall stress [4]. Global longitudinal strain (GLS) is a more sensitive measure of myocardial deformation and can detect subclinical systolic dysfunction even when LVEF is supranormal [13]. However, because both LVEF and GLS are afterload-dependent, they may overestimate effective LV performance or mask myocardial energetic inefficiency in oHCM. In this context, myocardial work analysis using non-invasive LV pressure–strain loops via speckle tracking echocardiography has emerged as a novel method to quantify the quality of LV contraction by integrating afterload into the assessment [16]. LV pressure–strain loop–derived indices provide incremental information beyond EF and strain alone, offering a measure of constructive vs. wasted work and overall myocardial efficiency [17]. Additionally, because the pressure–strain area reflects regional LV myocardial work, it also correlates with regional oxygen consumption. Recent developments have enabled estimation of systolic LV pressure (LVESP) curves non-invasively (using brachial cuff pressure and valvular event timing) to construct pressure–strain loops, a method originally validated against invasive pressure–volume measurements [16].

In this study, we applied non-invasive pressure–strain analysis to patients with oHCM before and under Mavacamten therapy. We aimed to determine whether Mavacamten optimizes myocardial work by reducing wasted work and improving myocardial work efficiency, consistent with the relief of outflow obstruction. We hypothesized that by lowering LVOT gradients and myocardial oxygen demand, Mavacamten would shift the pressure–strain loop parameters toward a more energetically favorable state, which could help explain the improvement in clinical status observed in real-world therapy.

## Methods

### Study population

We prospectively studied 42 patients with obstructive HCM enrolled in our institutional HCM registry, with a preliminary account of baseline echocardiographic and clinical data previously reported [18]. That prior report focused on real-world dose optimization, safety, and symptom response during the initiating phase of Mavacamten therapy. In the present analysis, we extended this cohort to evaluate the energetic consequences of Mavacamten using pressure–strain loop–derived myocardial work indices. Key inclusion criteria were diagnosis of HCM with LVOT obstruction (resting or provoked gradient ≥ 50 mmHg) and ongoing symptoms (NYHA class II or III) despite optimal conventional therapy. All patients had been on stable, maximally tolerated doses of β-blocker and/or verapamil for at least 4 weeks prior to enrolment. Exclusion criteria included LVEF < 50% and significant co-morbid cardiac conditions (e.g., moderate/severe valve disease other than mitral regurgitation secondary to HCM). All patients provided informed consent and were started on Mavacamten therapy between August 2023 and November 2024, according to the European Society of Cardiology (ESC) guideline recommendations [2]. Six patients were excluded from data analysis due to poor imaging quality or incomplete data acquisition. A total of 36 patients were included in the final study cohort. All patients underwent CYP2C19 metabolizer status testing before starting Mavacamten (as per European prescribing guidelines), and a starting dose of 5 mg daily was used in normal or intermediate metabolizers (2.5 mg in poor metabolizers). Mavacamten dosing was adjusted during the 12-week initiation phase if needed, based on LVOT gradient and LVEF monitoring, following a predefined protocol: doses were down-titrated to 2.5 mg if resting and provocable LVOT gradients fell below 20 mmHg, and up-titration to 10 mg was considered after 12 weeks for patients with persisting significant gradients (≥ 30 mmHg provoked) and symptoms, provided LVEF remained ≥ 50% [18]. No patient required discontinuation of Mavacamten due to excessive LV dysfunction or side effects during the study. Safety outcomes were defined as a drop in LVEF below 50%, cardiac hospitalization, and death.

### Echocardiographic assessment and pressure–strain analysis

**Fig. 1:**
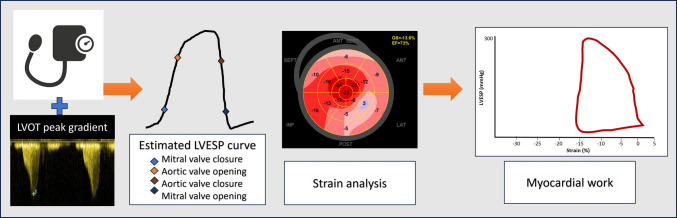
Pressure–strain loop analysis. Peak LVOT gradient is added to non-invasively measured blood pressure to obtain patients’ LV pressure curve and marked with valvular timing events (mitral valve closure, aortic valve opening, aortic valve closure, and mitral valve opening). An individualized LV pressure curve is plotted against LV strain analysis by speckle tracking, which generates a pressure–strain loop. The area within the LV pressure–strain loop (measured in mmHg% – the product of pressure and strain) is an index of myocardial work

Comprehensive transthoracic echocardiographic evaluations were conducted at baseline, after 12 weeks, and 12 months of Mavacamten therapy. Standard LV dimensions, wall thickness, LVEF, and left atrial (LA) diameter and volume were acquired using a GE Vivid E9 or E95 ultrasound system, and recordings were analyzed offline using the EchoPac workstation. The LVOT gradient was measured by continuous-wave (CW) Doppler at rest and during the Valsalva maneuver (provocation), with the higher value recorded as the peak provocable gradient. Mitral regurgitation severity was semi-quantitatively graded (none, mild, moderate) at each time point. Tissue Doppler and transmitral inflow were used to assess diastolic function (E/E′). For myocardial work analysis, we employed speckle-tracking echocardiography to obtain LV global longitudinal strain (GLS) from apical 4-, 3-, and 2-chamber views. E/E′, LA diameter, volume, and strain as well as LV biplane Auto-EF and global longitudinal strain were assessed to evaluate LA and LV function. After ensuring adequate image quality and frame rate (~ 60–80 fps), GLS was calculated as the average peak systolic longitudinal strain (%) of the 17 LV segments. Peak strain time dispersion (PSD) was also measured. To construct non-invasive LV pressure–strain loops, LVESP was estimated as systolic blood pressure plus peak LVOT gradient measured at rest by CW Doppler. This technique provides an estimated LV pressure throughout the cardiac cycle that, when plotted against myocardial strain, generates a pressure–strain loop for each cardiac cycle (Fig. 1). From the LV pressure–strain loop, we calculated the following global work indices for each patient:

1. Global work index (GWI) as described by Smiseth et al. [17], which measures the total work performed by the LV during one cardiac cycle, represented by the area within the LV pressure–strain loop (measured in mmHg%—the product of pressure and strain, an index of work). This reflects the total myocardial work, incorporating both constructive and wasted components.
2. Global constructive work (GCW), which reflects the work contributing to LV ejection. It quantifies as the work performed during shortening in systole plus the lengthening work during isovolumetric relaxation.
3. Global wasted work (GWW), which is the work performed during myocardial lengthening in systole (inefficient stretching of myocardium while the ventricle is generating pressure) and any work during shortening in isovolumic relaxation. This represents energy wasted against the LVOT obstruction or other non-productive forces.
4. Global work efficiency (GWE), which is an index of how efficiently the myocardium converts energy into useful work, calculated as GCW divided by the sum of GCW + GWW (GWE% = GCW/[GCW + GWW]). A higher GWE indicates that most of the cardiac work is constructive, whereas a lower GWE indicates a larger fraction of wasted work.

**Fig. 2:**
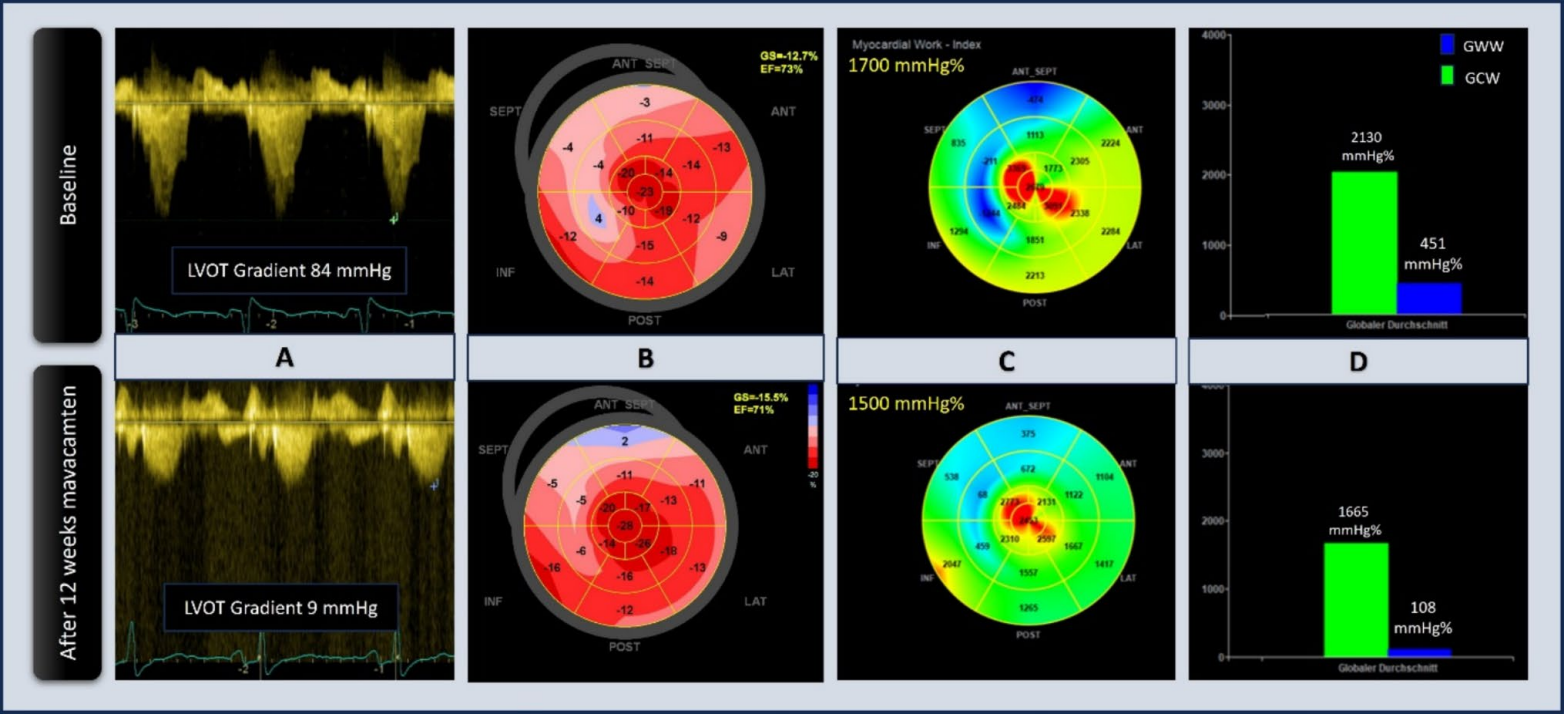
Case example of a patient with obstructive hypertrophic cardiomyopathy demonstrating echocardiographic changes after 12 weeks of Mavacamten therapy. **A** Resting peak LVOT gradients at baseline and after 12 weeks of Mavacamten therapy. **B** GLS at baseline and after 12 weeks of Mavacamten therapy. **C** Myocardial work index (GWI) at baseline and at 12 weeks after Mavacamten. **D** Global constructive work and global wasted work at baseline and at 12 weeks after Mavacamten. Abbreviations: LVOT, left ventricular outflow tract; GLS, global longitudinal strain; GWI, global work index

Figure 2 demonstrates these echocardiographic changes in a case example after 12 weeks of treatment with Mavacamten.

### Biomarkers and clinical outcomes

Venous blood samples were collected at baseline and at 12 weeks to measure high-sensitivity cardiac troponin I (hs-cTnI) and N-terminal pro–B-type natriuretic peptide (NT-proBNP), as indices of myocardial injury and wall stress, respectively. Patients’ functional status was assessed by New York Heart Association (NYHA) functional class and the Kansas City Cardiomyopathy Questionnaire (KCCQ) at both time points.

### Statistical analysis

Statistical analysis was performed using GraphPad Prism 8.1.2. Continuous variables are expressed as mean ± standard deviation or median (interquartile range) if not normally distributed. The Kolmogorov–Smirnov test was used to test the normality of the data. Categorical data are described as absolute numbers and percentages (%). Changes in outcome variables between baseline and the 3-month follow-up, and between the 3-month and 12-month follow-up, were assessed using Student’s *t*-test or the nonparametric Mann–Whitney *U* test, as appropriate. Pearson correlation analysis was used to examine the relationship between two variables. The statistical comparison of parameters above and below the median of GCW was performed using the *t*-test for normal distribution; otherwise, the nonparametric Mann–Whitney *U* test was used.

## Results

### Baseline characteristics

Thirty-six symptomatic patients with oHCM were included in the analysis. Baseline demographic and clinical characteristics are presented in Tables 1, 2, and 3. The cohort had a mean age of 61.2 ± 12.7 years (range, 30–87 years), with 19 patients being female (52.8%) (Table 1). All patients had a genetically confirmed or clinically diagnosed HCM with asymmetric septal hypertrophy. Baseline medications included β-blockers in 69.4% and verapamil in 16.7%; the remaining 13.9% were not on HCM-specific negative inotropic therapy due to prior intolerance or hypotension [18]. None of the patients had *CYP2C19* poor metabolic status. All were symptomatic (NYHA class II in 18 patients, class III in 18) despite medical therapy (Table 2). Six patients had previously undergone alcohol septal ablation (> 12 months prior) with persistent or recurrent LVOT obstruction, and eight patients had an implanted cardioverter defibrillator (ICD). The average basal interventricular septal thickness was 21.2 ± 3.4 mm, and the mean LVEF was 68 ± 6% (Table 3). Resting LVOT gradient averaged 69 ± 36 mmHg, and all patients had a provocable gradient ≥ 50 mmHg (provoked mean 113 ± 33 mmHg) (Table 3), confirming significant obstruction under both resting and physiological stress conditions.

**Table 1:** Baseline characteristics of patients initiated on Mavacamten

	*N* = 36
Mean age (years)	61.2
Sex	w 19/m 17
Height (cm)	170 ± 10
Weight (kg)	81.7 ± 20
History of septal reduction therapy	6 (16.7%)
Baseline medical therapy	
ß-blockers	25 (69.4%)
Verapamil	6 (16.7%)
None	5 (13.9%)
Atrial fibrillation	0
Presence of pacemaker/ICD	8 (22.2%)
*CYP2C19* metabolism: non-poor metabolizer	36 (100%)

Abbreviations: *NYHA* class, New York Heart Association functional class; *LVOT*: left ventricular outflow tract; *ICD*, implantable cardioverter defibrillator; *CYP2C19*, cytochrome P450 2C19 enzyme

**Table 2:** Clinical and safety parameters of 36 obstructive hypertrophic cardiomyopathy (oHCM) patients after 12 weeks of Mavacamten therapy

	Baseline	At 12 weeks	*p*-value
NYHA functional class			
I	0	19	
II	18	15	
III	18	2	
IV	0	0	
Blood pressure			
Systolic	131 ± 23	123 ± 21	
Diastolic	76 ± 11	76 ± 14	
KCCQ	52 ± 18	73 ± 20	< 0.0001
NTproBNP (ng/ml)	1495 ± 1391	570 ± 707	< 0.0001
Troponin (ng/mL)	28.5 ± 6.5	11.7 ± 5.6	< 0.001
Safety parameters			
Drop of LVEF < 50%	1		
Cardiac hospitalization	0		
Death	0		
Syncope	0		
Atrial fibrillation	0		

Abbreviations: *NYHA class*, New York Heart Association functional class; *LVOT*, left ventricular outflow tract; *LVEF*, left ventricular ejection fraction; *NT-proBNP*, N-terminal pro B-type natriuretic peptide; *LAVI*, left atrial volume index; *E/Eʹ*, ratio of early mitral inflow velocity to mitral annular early diastolic velocity

**Table 3:** Changes in key echocardiographic and myocardial work parameters in 36 obstructive hypertrophic cardiomyopathy (oHCM) patients after 12 weeks of Mavacamten therapy

	Baseline	At 3 months	*p*-value (baseline vs. 3 months)	At 12 months	*p*-value (3 vs. 2 months)
General echocardiographic parameters					
LVOT gradient at rest (mmHg)	69 ± 36	24 ± 27	< 0.001	11.4 ± 6	0.003
LVOT gradient under provocation (mmHg)	113 ± 33	50 ± 31	< 0.001	30.8 ± 19	0.01
IVSd (mm)	21.2 ± 3.4	19.9 ± 3.6	0.005	19.3 ± 3.7	0.15
LVEF (%)	68 ± 6	63 ± 5	0.003	62 ± 5	0.99
GLS (%)	−15.3 ± 3.6	−15.4 ± 3.0	0.9	−15.1 ± 2.8	0.41
E/E′	16.9 ± 5.8	14.4 ± 4.5	0.004	12.6 ± 5.3	0.005
LAVI (ml/m^2^)	40.9 ± 13.2	28.4 ± 14	0.08	34.9 ± 16.3	0.21
LA strain	22.5 ± 8.1	22.5 ± 6.3	0.99		
Myocardial work parameters					
GWI (mmHg%)	2098 ± 700	1610 ± 440	< 0.001	1538 ± 402	0.67
GWE (%)	89 ± 6	89 ± 6	0.71	88 ± 8	0.76
GCW (mmHg%)	2514 ± 776	1951 ± 466	< 0.001	1901 ± 380	0.74
GWW (mmHg%)	312 ± 163	249 ± 177	0.003	207 ± 124	0.30
PSD (ms)	116 ± 56	97 ± 36	0.07	93 ± 38	0.28

Abbreviations: *LVOT*, left ventricular outflow tract; *IVSd*, interventricular septum end-diastolic dimension; *LVEF*, left ventricular ejection fraction; *GLS*, global longitudinal strain; *E/Eʹ*, ratio of early mitral inflow velocity to mitral annular early diastolic velocity; *LAVI*, left atrial volume index; *GWI*, global work index; *GWE*, global work efficiency; *GCW*, global constructive work; *GWW*, global wasted work; *PSD*, peak strain time dispersion

NT-proBNP was elevated at baseline (1495 ± 1391 pg/mL), reflecting significant wall stress, and hs-cTnI was also elevated (28.5 ± 6.5 ng/mL), consistent with low-level chronic myocardial injury (Table 2). These findings underscore the hemodynamic burden and myocardial strain present before myosin inhibition therapy.

Baseline pressure–strain loop analysis revealed impaired myocardial efficiency compared to healthy reference values from F. J. Olsen et al. [19], who conducted a large-scale study of a healthy population consisting of 1827 participants (median age, 45 years). The average global work index (GWI) at baseline was 2098 ± 700 mmHg% (Table 3), comparable to normal values (2118 ± 277 mmHg% [19]), suggesting preserved total LV work per cardiac cycle. However, global wasted work (GWW) was markedly elevated (312 ± 163 mmHg%), far above the reference range (64 [47–89] mmHg% [19]), indicating substantial non-productive energy expenditure. Consequently, global work efficiency (GWE) was lower than normal: 89 ± 6% vs. 97.1% [96.0–97.9] in healthy subjects [19].

Notably, global constructive work (GCW) was elevated at 2514 ± 776 mmHg% (Table 3), when qualitatively elevated compared to reference ranges (2262 ± 283 mmHg% [17]), suggesting a much larger effective work in oHCM to overcome the high obstruction of the LVOT. In sum, prior to Mavacamten, patients expended a large share of their energy in non-productive work (overcoming the LVOT obstruction and related forces), reflected in high GWW and reduced GWE.

### Effects of Mavacamten on LVOT gradients, function, and clinical status

**Fig. 3:**
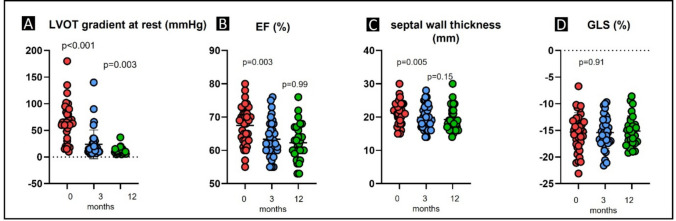
Hemodynamic changes and left ventricular function in 36 oHCM patients under Mavacamten therapy at 3 and 12 months. **A** resting LVOT gradient, **B** LVEF, **C** IVSd, and **D** GLS. Abbreviations: LVOT, left ventricular outflow tract; LVEF, left ventricular ejection fraction; IVSd, interventricular septum end-diastolic dimension; GLS, global longitudinal strain

All 36 patients completed the 12-week follow-up on Mavacamten, and 12-month follow-up data was available in 29 patients. The mean resting LVOT gradient fell from 69 ± 36 to 24 ± 27 mmHg, and the provoked LVOT gradient declined from 113 ± 33 at baseline to 50 ± 31 mmHg (*p* < 0.001 for both) (Fig. 3, Table 3). A further decrease was observed at 12 months (resting LVOT gradient, 11 ± 6 mmHg; provoked LVOT gradient, 31 ± 19 mmHg). This reduction in afterload translated into significant symptomatic improvement: by 12 weeks, 19 patients (52.8%) were NYHA class I, 15 were class II (41.7%), and the remaining 2 were class III. The KCCQ summary score improved from 52 ± 18 at baseline to 73 ± 20 (*p* < 0.0001) at 3 months, indicating substantial gains in quality of life [16]. These clinical improvements were accompanied by a significant decline in NT-proBNP (from 1495 ± 1391 to 570 ± 707 ng/mL; *p* < 0.0001) and hs-TnI (from 28.5 ± 6.5 to 11.7 ± 5.6 ng/mL; *p* < 0.001) (Table 2), consistent with reduced wall stress and injury. Furthermore, septal thickness (IVSd) and E/E′ ratio decreased significantly, while LAVI showed a non-significant trend toward reduction (Table 3).

LV systolic function, as measured by LVEF, declined slightly with Mavacamten (from 68 ± 6 to 63 ± 5% at 12 weeks and 62 ± 5% at 12 months; *p* = 0.003 for baseline to 3 months, *p* = 0.99 for 3 to 12 months), consistent with Mavacamten’s negative inotropic action, but remained above 60% in nearly all patients. Only one patient experienced a transient LVEF drop below 50%, prompting dose adjustment and successful continuation of therapy. Two patients discontinued treatment due to lack of efficacy, and one due to QT prolongation; these patients were not included in the study cohort.

Consistent with LVOT gradient reduction, mitral regurgitation improved by one grade in ten patients (27.8%), while the remaining 26 showed no change [18]. No patient developed worsening mitral regurgitation. There were no cardiac hospitalisations, deaths, syncope, or new-onset atrial fibrillation that occurred during the treatment phase. Safety and efficacy outcomes are listed in Table 2.

### Effects of Mavacamten on myocardial work indices

**Fig. 4:**
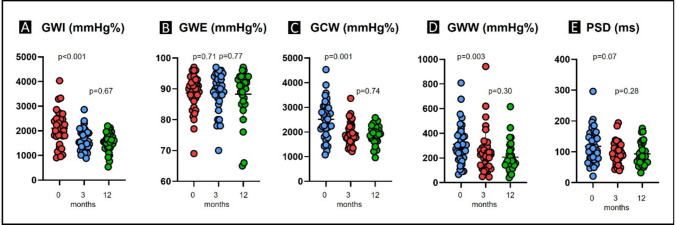
Myocardial work improvements after 3 and 12 months of Mavacamten therapy in oHCM patients. **A** Changes in GWI from baseline to 12 weeks under Mavacamten therapy. **B** GWE at baseline (red) and after (blue) 12 weeks of treatment with Mavacamten. **C** GCW at baseline (red) and after (blue) 12 weeks of treatment with Mavacamten. **D** GWW at baseline (red) and after (blue) 12 weeks of treatment with Mavacamten. **E** PSD at baseline (red) and after (blue) 12 weeks of treatment with Mavacamten. Abbreviations: GWI, global work index; GWE, global work efficiency; GCW, global constructive work; GWW, global wasted work

While GLS was unaltered (− 15.3 ± 3.6% vs. − 15.4 ± 3.0% at 3 months vs. − 15.1 ± 2.8% at 12 months; *p* = 0.9/0.41) (Table 3), peak strain time dispersion (PSD) showed a non-significant trend toward reduction after 3 and 12 months of Mavacamten therapy (from 116 ± 56 ms at baseline to 97 ± 36 ms at 3 months, *p* = 0.07, and to 93 ± 38 ms at 12 months, *p* = 0.28), indicating a potential improvement in synchronization of myocardial contraction (Fig. 4). The primary novel finding is that Mavacamten led to a marked improvement in myocardial energetics. Global wasted work (GWW) declined from 312 ± 163 to 249 ± 177 mmHg% (*p* = 0.003), corresponding to a ~ 20% reduction at 3 months, with a further decrease to 207 ± 124 mmHg% at 12 months (Table 3). Although GWW remained elevated vs. healthy references (64 [47–89] mmHg% [19]), this represents a meaningful reduction in non-productive energy expenditure. Constructive work (GCW) also decreased, from 2514 ± 776 to 1951 ± 466 mmHg% (*p* < 0.001) at 3 months and to 1901 ± 380 mmHg% (*p* = 0.74) at 12 months, reflecting lower LV pressure demands post-gradient reduction (Table 3). This post-treatment GCW approached healthy reference values (2262 ± 283 mmHg% [19]).

Total myocardial work (GWI) declined from 2098 ± 700 to 1610 ± 440 mmHg% (*p* < 0.001) at 3 months and to 1538 ± 402 mmHg% (*p* = 0.67) at 12 months, falling slightly below normal (2118 ± 277 mmHg% [19]) (Table 3). While a reduction in total work might initially be viewed as negative (less work done by the heart), in this context, it can be interpreted as favorable because the excess work was essentially pathological. Global work efficiency (GWE) remained unchanged (89 ± 6% at baseline and 3 months vs. 88 ± 8% at 12 months, *p* = 0.76) and below the healthy reference of 97.1% (96.0–97.9% [19]), suggesting that while absolute wasted and constructive work both declined, their ratio was preserved.

**Fig. 5:**
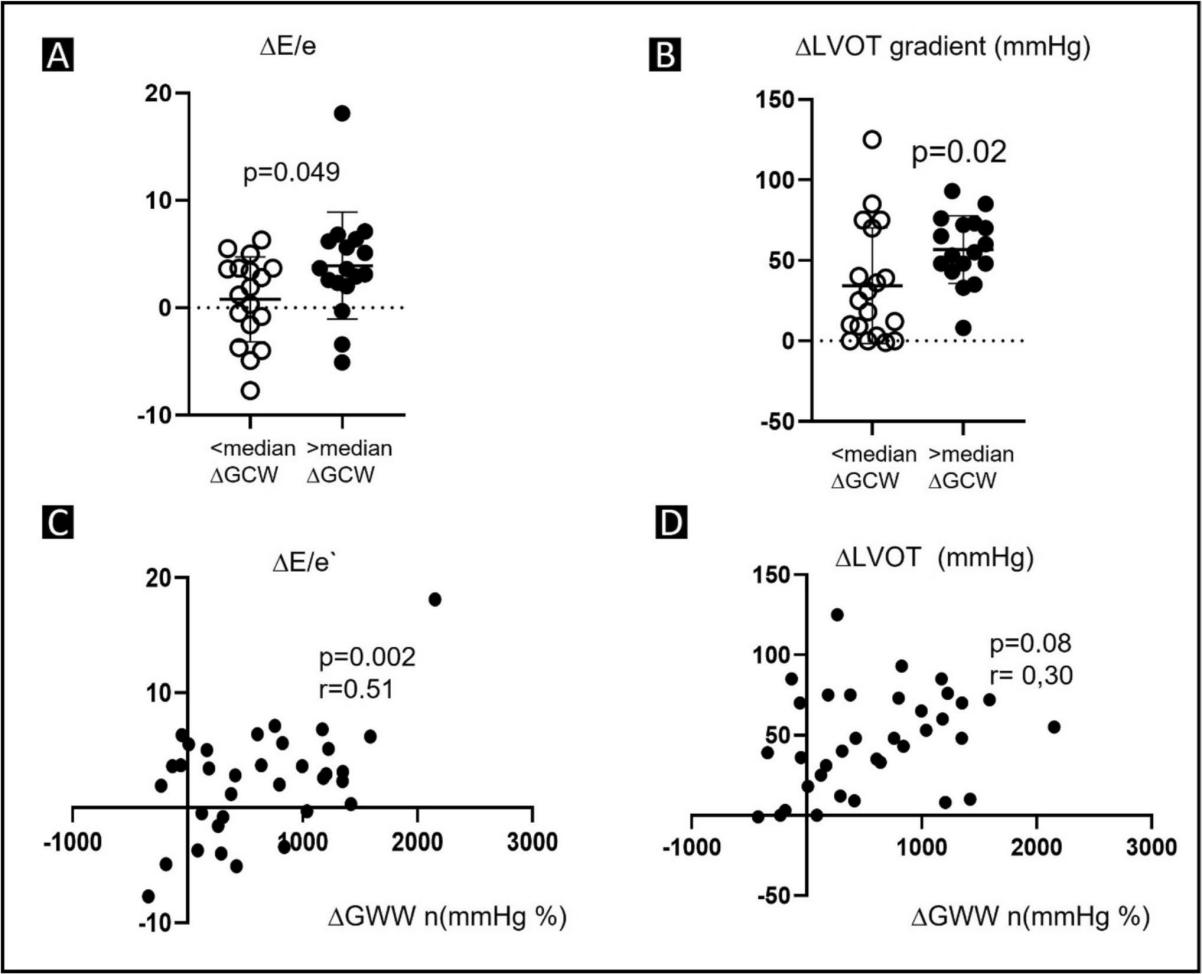
Association of changes in global constructive work with echocardiographic and biomarker parameters. **A** ΔE/E′ ratio in patients stratified by the median ΔGCW (open circles represent patients with ΔGCW < median and filled circles represent those with ΔGCW > median of 420 mmHg%. **B** Δ resting LVOT gradient in patients stratified by the median Δ GCW of 420 mmHg%. **C** Relationship between changes in ΔGCW and changes in diastolic filling pressure estimated by ΔE/E′ with a moderate and statistically significant correlation (*r* = 0.51, *p* = 0.002) and **D** changes in left-ventricular outflow tract velocity (ΔLVOT) showing a weak, non-significant association (*r* = 0.30, *p* = 0.08). Abbreviations: ΔE/E′, delta of the ratio of early mitral inflow velocity to mitral annular early diastolic velocity; Δ GCW, delta of global constructive work between baseline and 3 months; Δ LVOT, change of resting LVOT gradient between baseline and at 3 months

When patients were stratified by the change in GCW (median ΔGCW = 420 mmHg%), the cohort with a greater reduction in GCW (ΔGCW > 420 mmHg%) displayed significantly greater improvements in E/E′ (*p* = 0.049) and resting LVOT gradient (*p* = 0.02) compared to the group with a smaller reduction. The changes in NTproBNP were not significantly different between these groups (*p* = 0.17). Furthermore, the change in global work efficiency (ΔGCW) itself showed only a weak, non-significant association with the change in LVOT velocity (ΔLVOT; *r* = 0.30, *p* = 0.08). In contrast, ΔGCW correlated moderately and significantly with the improvement in diastolic function, as reflected by ΔE/E′ (*r* = 0.51, *p* = 0.002) (Fig. 5). No significant relationship was observed between ΔGCW and changes in NTproBNP levels (ΔBNP; *r* =  − 0.23, *p* = 0.16).

Taken together, these results indicate that Mavacamten reduces both productive and non-productive myocardial work, normalizing energy demand without altering mechanical efficiency. Improvements in diastolic parameters (E/E′ and LAVI) suggest reduced filling pressures, although unchanged LA strain may reflect persistent structural remodelling beyond the short treatment window.

## Discussion

### Energetic effects of Mavacamten: improving cardiac efficiency in oHCM

In this study, we provide new evidence on the energetic effects of Mavacamten therapy in oHCM, based on improvements in non-invasive myocardial work indices. The key finding is that Mavacamten not only reduces LVOT gradients and symptoms but also improves how efficiently the heart performs mechanical work. Before treatment, patients’ hearts exerted substantial internal effort to overcome outflow obstruction, a manifestation of the Anrep effect, where increased afterload leads to greater contractility and prolonged systole [4]. Our baseline data confirmed this: high total work, high wasted work, and reduced work efficiency, reflecting an energetically unfavorable state. After therapy, wasted work dropped significantly, and the global work index (GWI) improved, indicating a shift from unproductive to more useful cardiac work.

These effects can be understood in the context of LV pressure dynamics. In oHCM, systolic anterior motion of the mitral valve causes dynamic narrowing of the LVOT, such that ejection faces a rising resistance [3]. The LV must generate high intraventricular pressure to overcome this resistance, performing isovolumic work during ejection that does not contribute to forward flow. While stroke volume is preserved through the Anrep mechanism, this adaptation consumes excess energy [4, 5]. Reil et al. [4] confirmed this inefficiency with invasive pressure–volume loop data: oHCM patients had ~ 1.6-fold higher potential energy (unused energy) and ~ 50% mechanical efficiency, despite unchanged stroke volume post-septal ablation. In sum, it highlights how much energy the oHCM heart wastes in generating pressure rather than flow.

### Comparison to reference values and magnitude of change

To our knowledge, this is among the first studies to assess the effects of Mavacamten using pressure–strain loop–derived myocardial work indices. While prior EXPLORER-HCM [12] and VALOR-HCM [11] trials established clinical benefits, our results demonstrate corresponding energetic improvements. Wasted work fell by ~ 20%, and reductions in GCW and GWI (~ 600–700 mmHg%) were consistent with normalization toward reference values from healthy populations (e.g., 2118 ± 277 mmHg% for GWI and 2262 ± 283 mmHg% for GCW [19]). This supports the concept that pharmacological myosin inhibition can achieve comparable physiological effects through a non-invasive approach. Further support for the relevance of myocardial work assessment comes from cardiac resynchronisation therapy studies, where mechanical resynchronization was more likely when lateral-septal work differences exceeded 900 mmHg% [20].

### Clinical correlates and functional relevance

Our real-world findings align with clinical trial data showing symptom improvement and gradient reduction [18]. Patients experienced substantial improvements in NYHA class and quality-of-life scores (KCCQ), accompanied by reduced NT-proBNP and hs-TnI levels, consistent with less wall stress and myocardial injury. These biomarker improvements, combined with better energetic profiles, suggest potential for longer-term benefit (e.g., reduced fibrosis and disease progression), but this remains to be validated in extended studies.

Standard echocardiographic parameters such as LVEF and GLS showed minimal change, which is not unexpected. GLS is a more sensitive measure of myocardial deformation and can detect subclinical systolic dysfunction even when LVEF is elevated [13]. GLS also reflects contractility and is afterload dependent. Therefore, the negative inotropic effects of Mavacamten seem to be compensated by the reduced afterload following removal of the LVOT gradient, resulting in an almost unchanged GLS [14, 15].

### Methodological considerations in calculating myocardial work in oHCM patients

Estimating myocardial work requires an assumption about LVESP. In healthy individuals, LVESP is typically approximated by brachial systolic blood pressure. However, this assumption breaks down in oHCM, where dynamic intraventricular obstruction elevates LVESP beyond what is captured by peripheral pressure measurements. In fixed-valve conditions like aortic stenosis, LVESP can be approximated by adding systolic blood pressure to the mean transvalvular gradient [21, 22]. In oHCM, however, this equation is not directly applicable. The LVOT gradient varies (beat-to-beat) with changes in preload, afterload, and contractility, making the estimation more complex. Prior studies suggest that using the peak LVOT gradient, rather than the mean, more accurately reflects the true instantaneous afterload in this population [4, 23]. Although this method may overestimate absolute LVESP, it provides consistent and reproducible intra-individual comparisons across time points, which was the primary goal of our study.

The reductions in GCW, GWW, and GWI observed in our cohort are substantial in physiological terms. While global work efficiency (GWE) remained slightly below normal, the observed gains indicate that Mavacamten allows the oHCM heart to function under more favorable loading conditions. These findings reinforce a strong physiological rationale for using Mavacamten in symptomatic oHCM: it improves energetic efficiency, reduces wasted contractile effort, and potentially mitigates the long-term impact of elevated wall stress, including progression of hypertrophy, fibrosis, and systolic dysfunction.

## Limitations

This was a single-center study with 36 patients. Although the improvements in myocardial work were significant, larger multicenter trials with longer follow-up are needed to confirm these findings and evaluate long-term effects. The pressure–strain loop method used in this study estimates LV pressure curves non-invasively rather than relying on direct catheter-based measurements. LVESP was approximated by summing systolic blood pressure and the peak LVOT gradient for practicality. While this may slightly overestimate true LVESP, using each patient as their own control and observing stable blood pressure over time supports the reliability of relative changes. The method has shown strong correlation with invasive measures in other settings [16], and our findings align with established hemodynamic principles in oHCM [4], reinforcing its validity. We did not include a placebo or untreated control group due to ethical and practical constraints (symptomatic patients could not be denied effective therapy). Instead, a pre–post design was used, with comparisons to published healthy reference values (e.g., F. J. Olsen et al. [19]) providing additional context. Despite these limitations, our findings offer compelling support for Mavacamten’s role in improving cardiac efficiency and establish pressure–strain loop analysis as a practical, non-invasive tool for assessing treatment effects in oHCM. Future studies should integrate this technique to further explore myocardial mechanics in HCM and other high-afterload conditions.

## Conclusion

Mavacamten therapy reduces both the total and wasted myocardial work in patients with oHCM, reflecting a shift toward more favorable cardiac energetics. These effects likely contribute to the observed symptomatic and biomarker improvements and suggest that the benefits of Mavacamten extend beyond gradient relief to include meaningful changes in myocardial workload. Non-invasive pressure–strain loop analysis offers a valuable tool to quantify these changes and may inform future efforts to tailor therapy in HCM. Further studies are needed to determine whether the energetic improvements observed here translate into long-term reductions in disease progression and heart failure risk.

## Data Availability

The data underlying this article are anonymized and will be shared upon reasonable request to the corresponding author.

## Abbreviations

oHCM: Obstructive hypertrophic cardiomyopathy
LVOT: Left ventricular outflow tract
LVESP: Left ventricular end-systolic pressure
GLS: Global longitudinal strain
PSD: Peak strain time dispersion
GWI: Global work index
GCW: Global constructive work
GWW: Global wasted work
GWE: Global work efficiency
NYHA: New York Heart Association
NTproBNP: N-terminal pro B-type natriuretic peptide
SRT: Septal reduction therapy
